# Comparison of look-locker and MOLLI sequences for T1 mapping in hypertrophic and ischemic cardiomyopathy

**DOI:** 10.1186/1532-429X-15-S1-P78

**Published:** 2013-01-30

**Authors:** Xiaopeng Zhou, Melanie Traughber, Prabhakar Rajiah, Deborah Kwon, Paul Schoenhagen, Scott D Flamm, Randolph Setser

**Affiliations:** 1 Cleveland Clinic, Cleveland, OH, USA; 2 Cleveland State University, Cleveland, OH, USA; 3 Philips Healthcare, Cleveland, OH, USA

## Background

MOLLI gained popularity since it was first described in 2004, appearing to be more reliable to determine myocardial T1 time, but its relationship to LL, particularly in patients with myocardial disease, has not been fully evaluated. The purpose of this study was to compare pre- and post-contrast T1 values obtained from MOLLI and LL in several subject groups.

## Methods

5 control patients (4M/1F, age 44±21 y) with normal left ventricular (LV) function and no history of heart disease, 5 patients with ICM (3M/2F, 63±2 y), and 16 patients with HCM (10M/6F, age 51±15y) were recruited into an IRB approved protocol. All subjects were imaged at 1.5T (Achieva XR, Philips). T1 mapping was performed using MOLLI and LL at 2 LV short axis levels (basal- and mid-cavity) before and after contrast agent injection (Magnevist; 0.2 mmol/kg). Post gadopentetate dimeglumine (Magnevist; 0.2 mmol/kg) injection time was about 15 min for HCM and ICM patients and was shorter (about 5 min) for the controls due to organizational reasons. For each subject, TI measurements were made in 6 ROIs per level.

## Results

**Figure 1:**
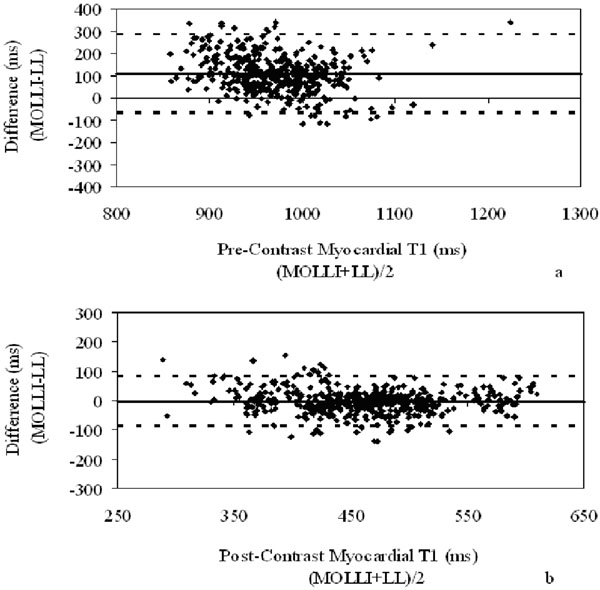
Bland-Altman analysis of myocardial T1 MOLLI vs. LL pre- (a) and post-contrast (b) injection in all patient groups.

Bland-Altman plots for all data combined are shown in Figure 1; trends were identical among the 3 groups when evaluated separately. Pre-contrast T1 values were significantly higher using MOLLI than LL for all 3 groups: controls (MOLLI 983±48ms vs LL 898±69ms, p<0.001), HCM (MOLLI 1029±44ms vs LL 917±58ms, p<0.001), and ICM (MOLLI 1083±136ms vs LL 900±112ms, p<0.001).

Post-contrast T1 values were significantly higher using MOLLI than LL for controls and HCM only: controls (MOLLI 474±70ms vs LL 465±64ms, p<0.001), HCM (MOLLI 488±59ms vs LL 474±54ms, p<0.001), and ICM (MOLLI 474±93ms vs LL 474±85ms, p=0.97).

## Conclusions

Pre-contrast MOLLI showed consistently higher T1 values in ICM and HCM patients compared to controls, which implies the potential application of pre-contrast T1 value in detecting the ICM and HCM. The elevation in T1 might be related to increased myocardial collagen content in HCM patients and impaired myocardial contractility in ICM patients. However, there was no similar T1 increase demonstrated with LL.

## Funding

This study was supported by Philips Healthcare and Cardiovascular Imaging Laboratory of Cleveland Clinic.

